# Identification of genes involved in the mutualistic colonization of the nematode *Heterorhabditis bacteriophora *by the bacterium *Photorhabdus luminescens*

**DOI:** 10.1186/1471-2180-10-45

**Published:** 2010-02-11

**Authors:** Catherine A Easom, Susan A Joyce, David J Clarke

**Affiliations:** 1 Department of Microbiology, University College Cork, Ireland; 2 Department of Biology and Biochemistry, University of Bath, BA2 7AY, UK

## Abstract

**Background:**

*Photorhabdus *are Gram negative entomopathogenic bacteria that also have a mutualistic association with nematodes from the family *Heterorhabditis*. An essential part of this symbiosis is the ability of the bacterium to colonize the gut of the freeliving form of the nematode called the infective juvenile (IJ). Although the colonization process (also called transmission) has been described phenomonologically very little is known about the underlying molecular mechanisms. Therefore, in this study, we were interested in identifying genes in *Photorhabdus *that are important for IJ colonization.

**Results:**

In this work we genetically tagged *P. luminescens *TT01 with *gfp *and constructed a library containing over 3200 mutants using the suicide vector, pUT-Km2. Using a combination of *in vitro *symbiosis assays and fluorescent microscopy we screened this library for mutants that were affected in their ability to colonize the IJ i.e. with decreased transmission frequencies. In total 8 mutants were identified with transmission frequencies of ≤ 30% compared to wild-type. These mutants were mapped to 6 different genetic loci; the *pbgPE *operon, *galE*, *galU*, *proQ*, *asmA *and *hdfR*. The *pbgPE*, *galE *and *galU *mutants were all predicted to be involved in LPS biosynthesis and, in support of this, we have shown that these mutants are avirulent and sensitive to the cationic antimicriobial peptide, polymyxin B. On the other hand the *proQ*, *asmA *and *hdfR *mutants were not affected in virulence and were either as resistant (*proQ*) or slightly more sensitive (*asmA, hdfR*) to polymyxin B than the wild-type (WT).

**Conclusions:**

This is the first report describing the outcome of a comprehensive screen looking for transmission mutants in *Photorhabdus*. In total 6 genetic loci were identified and we present evidence that all of these loci are involved in the assembly and/or maintenance of LPS and other factors associated with the cell surface. Interestingly several, but not all, of the transmission mutants identified were also avirulent suggesting that there is a significant, but not complete, genetic overlap between pathogenicity and mutualism. Therefore, this study highlights the importance of the cell surface in mediating the symbiotic and pathogenic interactions of *Photorhabdus*.

## Background

*Photorhabdus *are a genus of bioluminescent, entomopathogenic bacteria that are members of the family Enterobacteriaceae and are thus closely related to *Escherichia coli *and other important mammalian pathogens. As part of their normal life-cycle *Photorhabdus *also have a mutualistic interaction with nematodes from the family *Heterorhabditis *(for a recent review see [1]). The bacteria are normally found colonizing the gut of the infective juvenile (IJ) stage of the nematode. The IJ is the free-living infective stage of the nematode that is found in the soil and actively searches for potential insect larvae to infect. Once identified the IJ enters the insect through natural openings such as the mouth, anus or spiracles or the IJ can use a small tooth-like appendage to tear the cuticle and gain direct entry into the hemolymph. Once inside the insect the IJ migrates to the hemolymph where unidentified signals stimulate the IJ to regurgitate the bacteria. The bacteria avoid the insect immune response and grow exponentially within the insect until the insect succumbs to septicimeia within 48-72 h of infection [2]. At this point all of the internal organs of the insect have been converted into bacterial biomass. This bioconversion is facilitated by a range of hydrolytic enzymes that are secreted by *Photorhabdus*, including proteases and lipases. In the presence of high densities of *Photorhabdus *the IJ is stimulated to recover to a self-fertile adult hermaphrodite and this is the start of nematode reproduction. The hermaphrodite lays eggs and the developing nematode larvae feed on the bacteria present in the insect. As in *Caenorhabditis elegans*, the *Heterorhabditis *nematodes develop through 4 juvenile stages (J1-J4) before becoming adults [3]. Nematode reproduction continues for 2-3 generations until unidentified environmental stimuli triggers the formation of an alternative J3 nematode, the IJ, which exits the insect cadaver. Before leaving the insect cadaver the new IJ must be colonized by *Photorhabdus *and transmission of the bacteria to the IJ is a complex process that has only recently been phenomonologically described [4]. There are 2 striking features associated with the transmission process: 1) the colonization of the rectal gland cells of the adult hermaphrodite by *Photorhabdus *and 2) the observation that all IJs develop inside the adult hermaphrodite in a process called *endotokia matricida*. Therefore the bacteria that colonize the adult hermaphrodite are ultimately responsible for the colonization of the IJ [4].

The molecular mechanisms underlying the transmission process are poorly understood. In the only previous published study that reports a gene involved in transmission it was shown that a mutation in a gene annotated as *pbgE1 *severely affects the ability of *Photorhabdus *to colonize the IJ [5]. This mutant was isolated during a screen for genes affecting swimming motility and the *pbgE1 *mutant was also shown to be severely attenuated in virulence. The *pbgE1 *gene is predicted to be part of a 7 gene *pbgPE *operon that is homologous to the *arn *operon in *Salmonella *[5]. The *arn *operon has been shown to be involved in the modification of the lipid A moiety of LPS with L-aminoarabinose in response to the presence of cationic antimicrobial peptides (CAMPs) [6-8]. The *pbgE1 *mutant did produce altered LPS compared to the wild-type implicating LPS structure as a nematode colonization factor in *Photorhabdus *[5]. In this study we screened a library of *Photorhabdus *mutants with the aim of extending our understanding of the transmission process by identifying genes important in the colonization of the *H. bacteriophora *IJ nematode by *P. luminescens *TT01.

## Results

### Construction of a GFP-tagged strain of *P. luminescens *TT01

**Figure 1:**
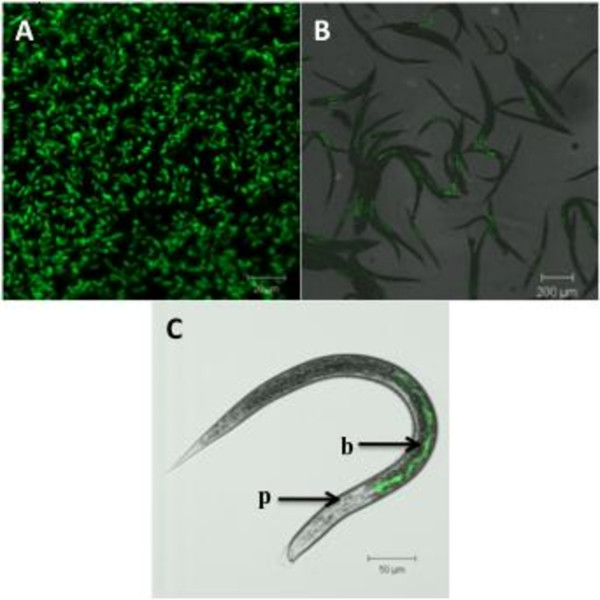
**Visualization of *P. luminescens *TT01_gfp _using fluorescent microscopy**. A) Image of a population of TT01_gfp _cells from a culture grown for 24 hours statically at 30°C; B) IJs colonized with TT01_gfp _(note that > 80% of the IJs can be seen to be colonized with TT01_gfp_); C) a fluorescent micrograph overlaid with a brightfield image of a single IJ confirming that the bacteria are located at the proximal end of the gut near the pharynx (p: pharynx; b: TT01_gfp_).

In order to facilitate a relatively rapid screening method for the identification of transmission mutants we decided to tag TT01 with a constitutively expressed *gfp *using a mini-Tn*7 *transposon (see Methods). This would enable the translucent IJs to be viewed beneath a fluorescent microscope and be scored qualitatively for the presence/absence of bacteria. Colonies of the *gfp*-tagged strain (called TT01_gfp_) were initially checked for fluorescence using a UV light box before overnight cultures were checked for *gfp *expression using a fluorescent microscope. This confirmed that the vast majority of cells in an overnight population of TT01_gfp _were expressing *gfp *(see Figure 1A). Phenotypic comparisons of TT01 and TT01_gfp _confirmed that there was no difference in growth rate, bioluminescence, pigmentation or virulence to insect larvae. Furthermore we also verified that TT01_gfp _was able to colonize IJ nematodes (see Figure 1B) with a transmission frequency identical to TT01 (between 80-85%). As has been previously shown, the TT01_gfp _bacteria were confirmed to occupy the proximal region of the nematode gut extending from just below the pharynx of the IJ (see Figure 1C).

### Identification of TT01_gfp _mutants affected in colonization of the IJ

**Figure 2:**
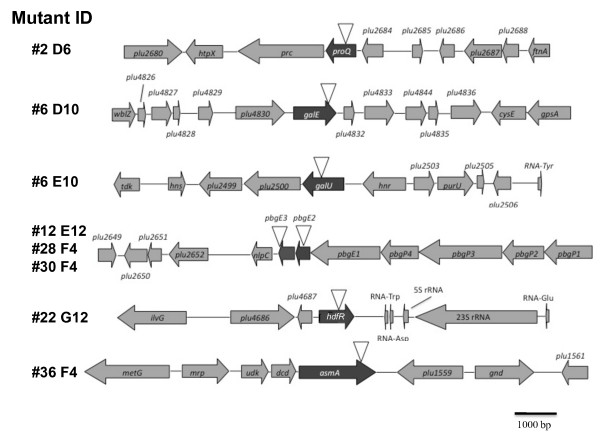
**The genetic loci important for colonization of the IJ**. The position of the transposon in each mutant was identified by sequencing and subsequent BLAST analysis using PhotoList http://genolist.pasteur.fr/PhotoList.

In this study we were using a qualitative screen that was designed to identify mutants that were affected in transmission frequency i.e. we were looking for mutants that colonized significantly fewer IJs than the 80% level observed with TT01_gfp_. Therefore TT01_gfp _was subjected to transposon mutagenesis using the Tn5 interposon delivered by plasmid pUT-Km2 and individual mutants were arrayed into 96 well plates and frozen. From this arrayed library 3271 mutants were screened for a defect in transmission frequency by growing the mutant on a lipid agar plate and inoculating the biomass with 30 surface-sterilized *H. bacteriophora *IJs. After 21 days incubation the new generation of IJs were collected and checked for colonization using a fluorescent microscope. In this way 40 mutants were identified as having a qualitative defect in transmission frequency i.e. <50% of the IJs were observed to be colonized by the mutant bacteria. Each mutant was then re-screened (in triplicate) and approximately 120 IJs in total from each mutant were individually examined using fluorescence in order to get a quantitative measure of transmission frequency. As a result we identified 10 mutants that reproducibly gave transmission frequencies of <35% (see Table 1). The gene that was interrupted in each mutant was identified (with the exception of #26 F7 and #32 F12) and the loci affected are shown in Figure 2. Mutants #12 E12 and #28 F4 were both identified as interuptions in *pbgE2*, a gene involved in LPS biosynthesis and part of an operon, the *pbgPE *operon, previoulsy identified as being important for IJ colonization [5]. The *pbgPE *operon is predicted to be involved in modification of the lipid A moiety of LPS with L-aminoarabinose. Interestingly we also identifed a mutation in the downstream gene, *pbgE3*, confirming a key role for this operon in IJ colonization. From this group of 3 mutants we used mutant #28 F4 for all further analysis. Mutants #6 D10 and #6 E10 were identified as interuptions of *galE *and *galU *respectively. The *galE *gene is predicted to encode UDP-glucose 4-epimerase and *galU *is predicted to encode glucose-1-phosphate uridyltransferase. Both of these activities are important in the production of polysaccharides including O-antigen [9-11]. Mutant #36 F4 was identified as an interuption of a gene with homology to the *asmA *gene in *E. coli*. The AsmA protein is localised to the outer membrane of *E. coli *and mutations in this gene resulted in significantly lower levels of LPS [12,13]. Mutant #22 G12 was identified as an interuption of a gene with homology to *hdfR *in *E. coli*. The *hdfR *gene has been shown to repress *flhDC *expression, and thus motility, in *E. coli *[14]. Finally mutant #2 D6 was shown to be an interuption of gene with homolgy to *proQ *from *E. coli*. In *E. coli proQ *encodes a protein that modifies the activity of ProP, a MFS transporter involved in the adaptation of the cell to osmotic stress [15,16]. However we could not identify a ProP homologue on the genome of TT01 suggesting a different role for ProQ in this bacterium.

**Table 1:** Colonization mutants identified in this study.

Mutant ID	Gene	Transmission frequency
#2 D6	*proQ*	27%
#6 D10	*galE*	31%
#6 E10	*galU*	23%
#12 E12	*pbgE2*	27%
#22 G12	*hdfR*	26%
#26 F7	nd	10%
#28 F4	*pbgE2*	30%
#30 F4	*pbgE3*	10%
#32 H12	nd	10%
#36 F4	*asmA*	20%

### Attachment of mutants to abiotic surfaces

**Figure 3:**
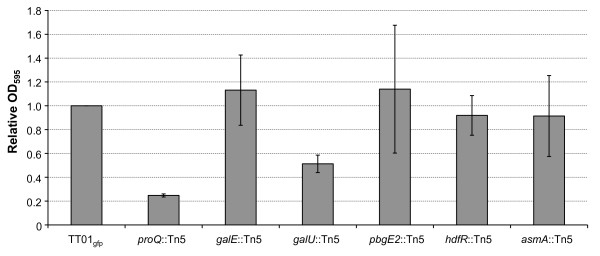
**Attachment to abiotic surface by *P. luminescens***. *Photorhabdus *strains (as indicated) were grown overnight at 30°C in LB broth (+ Km). The OD_600 _of the culture was adjusted to 0.05 and 200 μl was added to the well of a 96-well Costar^® ^PP microtitre plate. The plates were incubated for 72 h at 30°C before staining with crystal violet to quantify bacterial attachment. Relative biofilm formation was determined by calculating the OD_595 _(mutant):OD_595 _(TT01_gfp_) ratio and the results shown are the mean ± SD of 3 experiments.

Previous transmisson electron microscopy of *Photorhabdus *within the gut of the IJ had revealed features of the bacterial population that resembled growth as a biofilm i.e. the bacteria were seen to be in close association with the epithelial cells of the gut and encased in a matrix of unidentified composition [17]. Therefore we wanted to determine if any of the mutants defective in transmission to the IJ were affected in biofilm formation, as measured by attachment to an abiotic surface. The mutants were grown in the wells of a polypropylene (PP) microtitre plate for 72 h and the attached biomass was measured using crystal violet (see Figure 3). As can be seen only 2 mutants were affected in their ability to attach to PP, *proQ *and *galU *(20% and 45% of wild-type levels, respectively). All other mutants appeared to be unaffected in biofilm formation suggesting that colonization of the IJ and attachment to abiotic surfaces have distinct (yet overlapping) genetic requirements.

### Virulence of mutants to insect larvae

**Figure 4:**
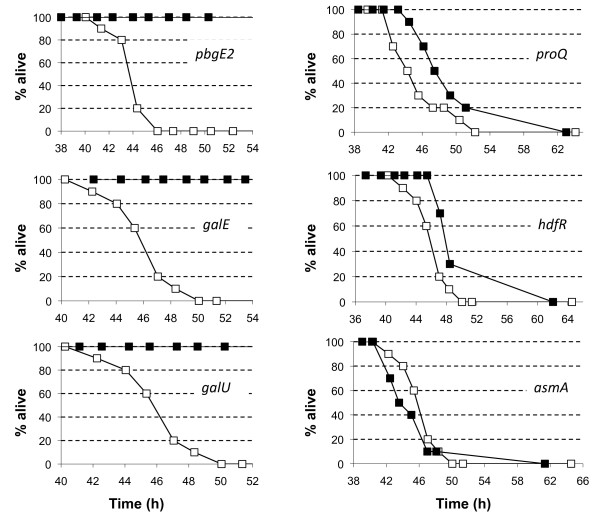
**Virulence of *P. luminescens *to insect larvae**. TT01_gfp _and mutant strains were grown overnight in LB broth at 30°C and diluted in PBS so that approximately 200 cfu were injected into each of 10 final-instar *G. mellonella *larvae. The insects were incubated at 25°C and insect death was monitored over the next 72 hours. In each graph the virulence of the mutant (■) is compared to TT01_gfp _(□). The results shown are of a representative experiment that was independently repeated at least 3 times.

*Photorhabdus *is highly virulent to insect larvae and previous work had shown that mutants affected in their ability to colonize IJs were also affected in their virulence to insects [5]. Therefore 200 cfu of each of the mutants was injected into 10 final instar larva of the Greater Wax Moth (*Galleria mellonella*) and insect death was assessed by gently prodding the insects at different time points post-infection. As expected the LT_50 _of TT01_gfp _was observed to be approximately 45-46 h (see Figure 4). This was similar to the LT_50_'s of the *proQ, hdfR *and *asmA *mutants suggesting that these genes are not important during virulence. We had previously shown that a mutation in the *pbgPE *operon was avirulent and this has now been confirmed in this study (see Figure 4). In addition the *galE *and *galU *mutants appeared to be completely avirulent under the conditions tested here implying an important role for polysaccharide production during virulence (see Figure 4).

### Sensitivity of mutants to polymyxin B

**Figure 5:**
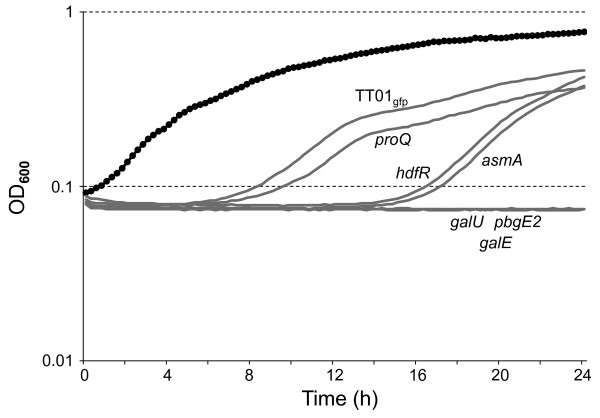
**Polymyxin sensitivity of *P. luminescens***. TT01_gfp _and mutant strains were grown overnight in LB broth and inoculated into fresh LB without (black curve) or with (grey curves) 2.5 μg ml^-1 ^polymyxin B (PB) added. The cells were grown for 24 h at 30°C and OD_600 _readings were taken every 15 mins. The growth curves of all of the strains were identical in the absence of added PB and therefore only a single representative curve (of TT01_gfp_) is shown. The growth curves of the strains growing in the presence of PB are labeled appropriately. Although the experiment was repeated 3 times only a representative growth curve of each mutant is presented.

Insects have a sophisticated innate immune system that includes the production of CAMPs [18]. One mechanism employed by bacteria to adapt to, and resist, the presence of CAMPs is to reduce the net negative charge associated with the LPS present in their outer membrane. This can be achieved by, amongst other means, replacing a negatively charged phosphate group on the lipid A moiety of the LPS with a positively charged L-aminoarabinose. In *Salmonella *and *E. coli *this modification is carried out by the products of the *arnBCADTFE *operon (formerly the *pmrHFIJKLM *operon) [7]. In *P. luminescens *the closest homologue to the *arnBCADTFE *operon is annotated as the *pbgPE *operon and we have previously shown that a mutation in this operon is hyper-sensitive to the presence of the CAMP, polymyxin B [5]. The *pbgPE *mutant is also avirulent to insects and unable to colonize the IJ suggesting a correlation between resistance to CAMP and the colonization of hosts [5]. To test this correlation further we analysed the ability of each of the mutants to grow in the presence of 2.5 μg ml^-1 ^polymyxin B. All of the mutants grew with the same growth rate as TT01_gfp _in LB broth without added PB (data not shown). However in the presence of polymyxin B the mutants could be divided into 3 groups based on the shape of their growth curve (see Figure 5). Both TT01_gfp _and the *proQ *mutant had very similar growth curves with a lag phase of approximately 5 h during which time it is likely that the cells are adapting to the prescene of the polymyxin B. The *hdfR *and *asmA *mutants were apparently slower to adapt and the lag time was extended to 14 h before the cells began exponential growth. Finally the *pbgE2*, *galE *and *galU *mutants did not show any growth in the presence of polymyxin B suggesting that these cells were unable to adapt to the presence of the CAMP.

## Discussion

In this study we screened over 3000 mutants of a *gfp*-tagged strain of *P. luminescens *TT01 for mutants that were reduced in their ability to colonize the guts of the IJ nematode i.e. transmission mutants. In total we identified 8 mutants in 6 different genetic loci: the *pbgPE *operon, *galE*, *galU*, *asmA*, *hdfR *and *proQ*. The transmission frequency of the identified mutants was between 10-30% indicating that none of these genes were required for colonization but, rather, somehow the genes improved the ability of the bacteria to colonize the IJ. Moreover, in those IJs that were colonized, the level of fluorescence observed suggested that the nematodes were carrying the full population of bacteria (data not shown). However we did not test for this directly by crushing and plating individual IJs.

The *pbgPE *operon is predicted to contain 7 genes, *pbgP1234pbgE123*, and in this study we identified mutations in both *pbgE2 *and *pbgE3 *that were affected in their ability to colonize nematodes. This work confirms an earlier study where we reported that a mutation in *pbgE1 *was important for both insect virulence and colonization of the IJ [5]. In this previous study we reported that the level of colonization of the *pbgE1 *mutant was 1% of the wild-type, in contrast to the 10-30% level reported here for the different mutations identified in the *pbgPE *operon (see Table 1). The reason(s) for this difference is not clear but it is nonetheless evident that the *pbgPE *operon plays an important role in the colonization of both the insect and the nematode.

In this study we demonstrated that mutations in *galU *and *galE *were affected in their ability to colonize the IJ. These genes are predicted to be involved in the biosynthesis of UDP-glucose and UDP-galactose, respectively, important precursors in the production of polysaccharides. The *galU *gene is predicted to encode glucose-1-phosphate uridyltransferase and is required for the production of UDP-glucose, an important glucosyl donor in the cell. In *Salmonella *UDP-glucose is required for the production of UDP-arabinose which is used to synthesise L-aminoarabinose for the modification of lipid A in response to CAMPs [19]. We have shown that the *galU *mutant does phenocopy the *pbgE2 *mutation suggesting that the *galU *defect may be explained by the associated defects in L-aminoarabinose biosynthesis. However we have also shown that, in contrast to the *pbgE2 *mutant, the *galU *mutant is defective in attachment to abiotic surfaces (see Figure 3) suggesting that the *galU *mutation is pleitropic. Indeed, in *E. coli*, a mutation in *galU *would also be expected to prevent production of the LPS-associated O-antigen [20]. In addition to LPS synthesis, UDPglucose also plays a role in protecting *E. coli *against thermal and osmotic shocks (through the production of trehalose and membrane-derived oligosaccharides (MDO)) and the negative regulation of σ^S^, the stationary-phase sigma factor [21,22].

However we have shown that σ^S ^is not required for either virulence or IJ colonization by *P. luminescens *(R. J. Watson and D. J. Clarke, unpublished data) implying that UDP-glucose is important in colonization through its role in polysaccharide biosynthesis. The *galE *gene is predicted to encode UDP-glucose-4-epimerase, an enzyme responsible for the interconversion of UDP-glucose and UDP-galactose. *P. luminescens *does not catabolise galactose (our unpublished data) suggesting that the main role of GalE is in the production of UDP-galactose from UDP-glucose. In *E. coli *both *galE *and *galU *are required for the production of LPS O-antigen [10] and, although the structure of the O-antigen is not known in *Photorhabdus*, it seems plausible that both UDP-glucose and UDP-galactose will be required for O-antigen biosynthesis. Indeed, given that the *galU *and *galE *mutants in *P. luminescens *are both avirulent to insects, sensitive to CAMPs and defective in colonization of the IJ, it seems likely that these mutants are affected in the same pathway i.e. LPS biosynthesis. Nonetheless it is interesting to note that, in contrast to the *galU *mutant, the *galE *mutant is not affected in attachment to an abiotic surface (see Figure 3). However this can be simply explained if, as expected, mutations in *galE *and *galU *(i.e. UDP-glucose and UDP-galactose) result in slightly different LPS molecules.

Our identification of mutants in *pbgPE*, *galE *and *galU *clearly implicates LPS as an important player in the colonization of the IJ by *Photorhabdus*. In this study we also identified mutations in genes that were not directly associated with LPS metabolism; *asmA*, *hdfR *and *proQ*. The *asmA *gene was originally identified in *E. coli *as a site for suppressor mutations of an assembly defective porin, OmpF315 [23]. Although the role of AsmA is still not clear it is likley that this protein is involved in organising the outer membrane. In the first instance a mutation in *asmA *has been shown to result in reduced levels of LPS in the outer membrane of *E. coli *[12]. In addition a recent study reported that a mutation in *asmA *in *Salmonella enterica *serovar Typhimurium resulted in a remodelling of the outer membrane that resulted in an increase in the transcription of *marAB*, encoding a multi-drug efflux pump [24]. The authors further report that the *S. enterica asmA *mutant was attenuated in virulence when administered orally to mice and showed a reduced ability to invade epithelial cells thus linking *asmA *with infection [24]. The *hdfR *gene was originally annotated as 2 overlapping genes, *yifA *and *pssR*, on the *E. coli *genome but recent analysis confirmed the presence of a sequencing error that resulted in a frameshift and the subsequent mis-annotation [14,25]. The *hdfR *gene is predicted to encode a LysR-type regulator that represses the expression of *flhDC*, and therefore motility, in *E. coli *[14]. In *Proteus mirabilis *2 independent mutations in *hdfR *were identified in a STM experiment as being important for urinary tract colonization in mice [26]. Motility has been shown to play an important role in *P. mirabilis *virulence however a role for *hdfR *in regulating motility in *Proteus *has not been determined [27]. Interestingly we have shown that the *hdfR *mutant does not appear to affect swimming motility in *P. luminescens *(data not shown). Finally we identified a mutation in the *proQ *gene. This gene is predicted to encode a protein that, in *E. coli*, is involved in the post-translational activation of ProP, an osmoprotectant/proton symporter that is capable of transporting both proline and glycine betaine in response to increases in osmotic pressure [15,16]. However the genome of *P. luminescens *is not predicted to encode a ProP homologue suggesting an alternative role for ProQ in *Photorhabdus*.

Interestingly the *proQ *mutant was the most affected in attachment to an abiotic surface suggesting alterations in the cell surface of the mutant (see Figure 3). However the *proQ *mutant was not sensitive to CAMPs suggesting that the LPS was not affected (see Figure 5). It is also noteworthy that, unlike the other mutants identified in this study, there is the possibility that the mutation in *proQ *has a polar affect on the downstream gene, *prc *(see Figure 2). The *proQ *and *prc *genes are separated by 20 bp on both the *E. coli *and *P. luminescens *genomes and *proQ *and *prc *are predicted to be on the same transcription unit in *E. coli *http://ecocyc.org. The *prc *gene encodes a periplamsic protease called Prc or Tsp (tail-specific protease) that processes the C-terminus of FtsI (PBP3) and is required for protection from combined osmotic and thermal stress [28,29]. Moreover Prc has been shown to interact with NlpI, a lipoprotein that has recently been shown to be involved in the attachment of adherent-invasive *E. coli *(bacteria associated with Crohns disease) to epithelial cells [30,31]. In addition, in *Pseudomonas aeruginosa*, Prc has been implicated in the regulation of alginate production by degrading mutant forms of MucA, the anti-sigma factor that interacts with the alternative sigma factor AlgU [32]. Therefore a decrease in the level of *prc *transcription may affect the surface of *Photorhabdus *in a way that prevents colonization of the IJ. However further experimentation is required to determine whether the *proQ *or *prc *gene (or both) are responsible for the reported phenotype.

## Conclusion

We have identified 5 genetic loci in *P. luminescens *TT01 that are affected in their ability to colonize IJs of the nematode *H. bacteriophora*. In order to have a reduced transmission frequency it would be expected that the mutants would be affected in either their ability to infect and replicate within the adult hermpahrodite or in their ability to colonize the IJ. Preliminarly studies, using confocal laser scanning microscopy (CLSM), suggest that all of the mutants are able to infect the adult hermaphrodite (our unpublished data). Therefore the defect in colonization appears to occur at some point later during the transmission process. It has been shown that colonization of the IJ requires binding to the pre-intestinal valve cell in the immature IJ followed by growth and replication of the bacteria in the gut lumen [4]. All of the mutants identified in this study can be implicated in the maintenance of the structure and/or remodelling the bacterial cell surface and it is, therefore, easy to envisage how mutations affecting the cell surface of *P. luminescens *could affect how the bacteria interact with the IJ. The exact stage and nature of the colonization defect of each mutant is currently under examination.

## Methods

### Bacterial strains and culture conditions

All *P. luminescens *strains were cultured in LB broth or on LB agar (LB broth plus 1.5% (w/v) agar) at 30°C. Unless otherwise stated all LB agar plates were supplemented with 0.1% (w/v) pyruvate. When required antibiotics were added at the following concentrations: ampicillin (Ap), 100 μg ml^-1^; chloramphenicol (Cm), 20 μg ml^-1^; gentamycin (Gm), 20 μg ml^-1^; kanamycin (Km), 25 μg ml^-1^and rifampicin (Rif), 50 μg ml^-1^.

### Construction of *gfp*-tagged *P. luminescens *TT01

A *gfp*-tagged strain of *P. luminescens *TT01 was constructed using the Tn7-based vector, pBKminiTn7-*gfp2 *[33]. Overnight cultures of *P. luminescens *TT01 (the recipient), *E. coli *S-17 (λpir) carrying pBKminiTn7-*gfp2 *(the donor) and *E. coli *carrying the helper plasmid pUXBF13 (the helper) were prepared in LB broth supplemented with 10 mM MgCl_2 _(MgLB). The cultures were diluted to OD_600 _= 0.05 in 20 ml of MgLB and grown, with shaking, at 30°C. The cells were harvested at an OD_600_= 0.5 and mixed in a 4:1:1 (v/v/v) ratio (recipient:donor:helper) in MgLB. The mixture (total volume = 300 μl) was added to the centre of a MgLB agar plate and incubated at 30°C overnight. The following day the cells were resuspended in MgLB, plated out onto MgLB_Rif Cm _agar plates and incubated at 30°C for 48 h. Cm^R ^Amp^S ^Km^S ^exconjugants were selected and colony PCR was used to confirm that the Tn*7 *and *gfp *had inserted in the expected position on the TT01 chromosome. The strain successfully tagged with *gfp *was renamed TT01_gfp_.

### Generating a mutant bank via Tn5 transposon mutagenesis

The Tn*5 *mutants were generated by conjugating TT01_gfp _with *E. coli *S17-1 (λ*pir*) carrying the suicide vector, pUT-Km2, as previously described [34]. In addition to expressing *gfp*, the Tn7 inserted into the chromosome of TT01_gfp _also confers resistance to both Cm and Gm. Therefore exconjugants were selected on LB _Rif Cm Km _and colonies were inoculated into 1.5 mls of LB_Cm Km _in each well of a 96 deep well plate, sealed with a gas permeable seal (Thermo scientific), and incubated overnight at 30°C. A 75 μl aliquot from each well was mixed with 75 μl of 40% (v/v) glycerol in a 96 well plate (Sterilin), sealed with an aluminium seal (Sarstedt), and frozen at -80°C.

### Screening for IJ colonization mutants

The nematode is translucent thus enabling visualization of TT01_gfp _within the gut of the IJ using fluorescence microscopy (see Figure 1). 50 μl of an overnight culture of each TT01_gfp_::Tn*5 *mutant was used to inoculate lipid agar supplemented with Rif, Cm and Km. Plates were incubated at 30°C for 48 h before 30 surface-sterilized *H. bacteriophora *IJs were added to each plate [5,35]. Symbiosis plates were incubated at 25°C for a minimum of 21 days. Next generation IJs were then washed from the surface of the Petri dish lids using 1 × PBS. An epifluorescent microscope, using blue light to excite *gfp *and white light to estimate number of IJs present, was used to qualitatively determine the percentage of IJs colonised in each well compared to a TT01_gfp _control (see Figure 1). Mutants qualitatively determined to have a transmission frequency < 50% were re-tested in triplicate. For a more quantitative estimation of transmission frequency the IJs were washed from the surface of the Petri dish lids and 10 IJ were taken, in quadruplicate, from each symbiosis plate and aliquoted into a 96 well flat-bottomed microtitre plate. Each mutant was therefore represented by 12 wells in the 96 well plate and, using epifluorescence microscope, the percentage colonization (i.e. transmission frequency) was determined per well and an average calculated for each TT01_gfp_::Tn*5 *mutant.

### Attachment to an abiotic surface

The capacity of *P. luminescens *to form biofilms was assessed by measuring bacterial attachment to a plastic surface, as previously described [34]. Briefly strains were grown overnight in LB broth, diluted to OD_600_= 0.05 in fresh LB and 200 μl of the cell suspension was aliquoted in triplicate, into the wells of a Costar^® ^polypropylene (PP) 96-well microtitre plate. The plates were sealed with a gas permeable membrane and incubated, without shaking, at 30°C. At the appropriate time the planktonic cells were removed by aspiration and 250 μl of 0.1% (w/v) crystal violet (CV) was added to each well. The plates were incubated at room temperature for 20 min before rinsing 3 times with 1 × PBS. To quantify biofilm formation the CV was dissolved in 250 μl of 95% ethanol and the CV concentration was determined by measuring the OD_595 _using a Genios (Tecan) plate reader.

### Pathogenicity assays

The pathogenicity of *P. luminescens *was assessed using *Galleria mellonella *larvae, purchased from Livefood (UK), as the model insect host. Briefly overnight cultures of *P. luminescens *TT01 were washed 3 times in 1 × PBS before the OD_600 _was adjusted to 1.0 (equivalent to 4 × 10^8 ^cfu ml^-1^). The culture was diluted with 1 × PBS and 10 μl (equivalent to 200 cfu) was injected into the hemolymph of a *G. mellonella *larva using a Hamilton syringe and a BD Microlance™ 3 30 G × 1/2" needle.

### Polymyxin sensitivity

To test for sensitivity to polymyxin B overnight cultures of each strain were diluted to an OD_600 _= 0.05 in either fresh LB or LB with 2.5 μg ml^-1 ^of freshly prepared polymyxin B (Sigma). From these dilutions 100 μl of each culture was inoculated, in triplicate, into wells of a 100 well Isotron honeycomb 2 plate. The plates were loaded into the Bioscreen C plate reader programmed to incubate the plates at 30°C and to take an OD_600 _reading every 15 minutes over a period of 24 hours.

## Authors' contributions

CAE undertook all of the experiments described in this manuscript. DJC, CAE and SAJ conceived of the study and designed the experiments and DJC drafted the manuscript. All authors read and approved the final manuscript.
